# Impact of molasses and microbial inoculants on fermentation quality, aerobic stability, and bacterial and fungal microbiomes of barley silage

**DOI:** 10.1038/s41598-020-62290-7

**Published:** 2020-03-24

**Authors:** Beiyi Liu, Zhiqing Yang, Hailin Huan, Hongru Gu, Nengxiang Xu, Chenglong Ding

**Affiliations:** 1 Institute of Animal Science, Jiangsu Academy of Agricultural Science, Nangjing, 210014 China; 2 Key Laboratory of Crop and Animal Integrated Farming Ministry of Agriculture, Jiangsu Academy of Agricultural Science, Nangjing, 210014 China; 3 Jiangsu Coastal Area Institute of Agricultural Sciences, Yancheng, 224002 China

**Keywords:** Applied microbiology, Microbial ecology

## Abstract

This study aimed to investigate the effects of microbial inoculants (L) and molasses (M) on the bacterial and fungal microbiomes of barley silage after the aerobic stage. The addition of molasses and microbial inoculants improved the aerobic stability of barley silage. The ML silage, which had a low pH value and high lactic and acetic acid contents, remained aerobically stable for more than 216 h. The ML silage exhibited low bacterial and high fungal diversities. Microbial inoculants and molasses enriched the abundance of *Lactobacillus* in silage after aerobic exposure. The enrichment of *L. buchneri* was significant in ML silage at days 5 and 7 during the aerobic stage. The abundance of harmful microorganisms, such as aerobic bacterial including *Acinetobacter*, *Providencia*, *Bacillus*, and yeasts including *Issatchenkia*, *Candida*, and *Kazachstania*, were suppressed in ML silage. M and L had an impact on bacterial and fungal microbes, resulting in the improvement of fermentation quality and reduction of aerobic spoilage in barley silage.

## Introduction

The growth and reproduction of aerobic bacteria (AB), yeasts, and molds usually lead to the poor fermentation quality of silage upon aerobic exposure^1^. Selective additives, such as lactic acid bacteria (LAB), are used to reduce aerobic spoilage and the accumulation of toxic matter in the progress of ensiling^2^. In our previous study, we investigated the effects of LAB on bacterial and fungal microbiomes during aerobic exposure and found that LAB inoculants improved the aerobic stability of barley silage, but the pH value was 4.85 after 7 d of aerobic exposure. *Issatchenkia* was the main microorganisms that caused aerobic spoilage in barley silage; however, the addition of LAB has little effect on fungal communities^3^. The use of silage additives individually or in combination during ensiling should reduce decomposition and prolong the stability of barley silage upon air exposure.

Molasses (M) is used as a fermentation stimulant to increase the rate of silage acidification by providing fermentable sugars for the growth of LAB during the ensiling of silage^4^. Studies have reported that the addition of M results in reducing pH values and increasing lactic acid (LA) concentrations in silages^5,6^. Several studies have focused on the effects of additives or M on fermentation characteristics and aerobic stability^7^, whereas others have examined silage quality and the dynamics of microbial community^8^. The addition of molasses had no pronounced effects on fermentation quality of alfalfa silage, but increased aerobic stability^9^. The simultaneous application of *L. plantarum* inoculant and molasses improved fermentability and aerobic stability of sawdust-based spent mushroom substrate silages^10^. However, few studies have comprehensively evaluated fermentation quality, aerobic stability, and microbial community dynamics in barley silage prepared with M and LAB after aerobic exposure, which may provide important data regarding aerobic stability of barley silage.

Microbial communities are essential to conserve barley silage. Next-generation sequencing (NGS) is an approach for the characterization and composition of microbial communities of silage^11^. Moreover, the composition and shift of microorganisms, including bacteria and fungi, determines fermentation quality and aerobic stability of barley silage. Hence, the identification of microbial shifts in barley silage with the addition of M and LAB is essential.

Therefore, NGS high-throughput sequencing was used in this study to analyze silages characteristics and bacterial and fungal microbiomes of barley silage with the addition of M and LAB during aerobic stage.

## Results

### Effect of ML on quality of barley silage during aerobic stage

**Figure 1:**
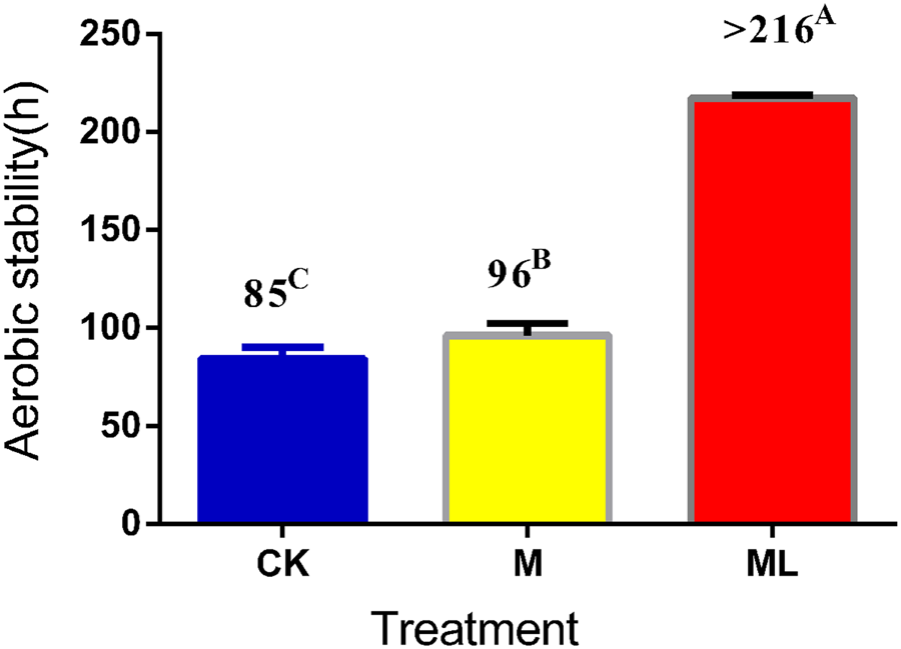
Effect of ML on aerobic stability of barley silage ensiled for 60 days. Data are presented as means of three replicates. Values in the same row (**A–C**) with different superscripts are significantly different (*p* < 0.05). Abbreviations: CK, control; M, molasses; ML, molasses and microbial inoculants.

The pH values of the control (CK), M silage and ML silage were within the range of 3.87–3.94 after 60 days of ensiling (Table 1). When exposed to air, the pH values of the CK and M silages increased rapidly with prolonged exposure but not of the ML silage. Changes in pH values were relatively constant in barley silage prepared with ML from day 0 to 7 of aerobic exposure. Similarly, the concentration of fermentation products, such as LA and AA, decreased gradually with prolonged exposure in the CK and M silage but not in the ML silage. Changes in the concentrations of fermentation products were significant during aerobic exposure of barley silage (*p* < 0.05). LA and AA contents were higher in the ML silage than the CK and M silages at days 2, 5, and 7 of aerobic exposure (*p* < 0.05). In addition, as shown in Fig. 1, the ML silage was aerobically stable for more than 216 h, whereas the CK and M groups were stable for only 85 h and 96 h, respectively, indicating that the aerobic stability of barley silage was improved by the addition of ML. The single addition of M minimally improved aerobic stability of silage. Although yeast and AB counts increased gradually with prolonged aerobic exposure, the LAB counts did not significantly decrease in ML silage (Table 2). Compared with the CK silage, the counts of LAB, yeast, and AB in barley silage prepared with M only changed significantly after 5 and 7 d of exposure (*p* < 0.05). Nevertheless, the yeast and AB counts of the ML group were significantly lower than those of the CK and M groups during aerobic exposure (*p* < 0.05).

**Table 1:** Effect of ML on pH, fermentation products of barley silage during aerobic exposure.

Items^1^	Treatments	Aerobic exposure days				SEM	P Value		
		0	2	5	7		D	A^3^	A × D
pH	CK^2^	3.94^aC^	4.00 ^C^	6.02^aB^	6.56 ^aA^	0.076	<0.001	<0.001	<0.001
	M	3.89^bB^	4.03^B^	5.53 ^aA^	5.78 ^aA^				
	ML	3.87^bB^	3.95^AB^	3.97^bAB^	3.98^bA^				
LA(%)	CK	5.07 ^aA^	3.38^bB^	2.56^bC^	1.61^bD^	0.034	<0.001	<0.001	<0.001
	M	4.87^abA^	3.32^bB^	1.89^cC^	1.68^bC^				
	ML	4.55^bC^	5.47^aB^	6.12 ^aA^	5.48^aB^				
AA(%)	CK	1.83^bA^	1.21^bB^	1.08^bB^	0.84^bC^	0.022	<0.001	<0.001	<0.001
	M	1.77^bA^	1.30^bB^	1.02^bBD^	0.98^bCD^				
	ML	3.04 ^aA^	1.88^aC^	2.19^aB^	2.27^aB^				
PA(%)	CK	0.31^b^	0.36^b^	0.41^b^	0.38^b^	0.011	0.007	<0.001	0.017
	M	0.42^abBC^	0.45^bBC^	0.52^abAB^	0.39^bC^				
	ML	0.52^aC^	0.66^aAB^	0.63^aBC^	0.77 ^aA^				
Et(%)	CK	2.25 ^aA^	1.34 ^C^	1.49 ^C^	1.53^BC^	0.019	<0.001	0.033	0.069
	M	1.88^bA^	1.42^B^	1.41^B^	1.44^B^				
	ML	1.95^bA^	1.42^CD^	1.33^D^	1.51^BC^				

Data are presented as means of three replicates. Values in the same row (A–D) or in the same column (a–c) with different superscripts are significantly different (p < 0.05).

^1^LA, Lactic acid; AA, Acetic acid; PA, Propionic acid; Et, Ethanol.

^2^CK, control; M, molasses; ML, molasses and microbial inoculants.

^3^A, Additive; D, ensilage time; A × D, the interaction between additive and ensilage time.

**Table 2:** Effect of ML on microbial counts and chemical composition of barley silage during aerobic exposure.

Items^1^	Treatments	Aerobic exposure days				SEM	P Value		
		0	2	5	7		D	A^3^	A×D
LAB (Log_10_cfu/g FM)	CK^2^	6.69^bA^	6.07^cB^	5.37^cC^	4.90^cD^	0.016	<0.001	<0.001	<0.001
	M	7.27 ^aA^	7.30^bA^	6.72^bB^	5.88^bC^				
	ML	7.37^aB^	7.99 ^aA^	7.33^aB^	6.99^aC^				
Yeast (Log_10_cfu/g FM)	CK	3.04^aC^	5.26^aB^	6.14 ^aA^	6.32 ^aA^	0.017	<0.001	<0.001	<0.001
	M	2.88^aD^	4.22^bC^	5.42^bB^	5.97^bA^				
	ML	2.10^bD^	3.31^cC^	4.26^cB^	4.71^cA^				
AB (Log_10_cfu/g FM)	CK	2.58^aC^	2.85^aC^	4.20^aB^	5.65 ^aA^	0.029	<0.001	<0.001	<0.001
	M	2.41^acD^	2.86^aC^	3.80^bB^	5.17^bA^				
	ML	2.14^bcC^	2.28^bC^	2.84^cB^	3.80^cA^				
DM (g/kg)	CK	333.67 ^A^	321.33^B^	308.33^bC^	295.67^bD^	0.708	<0.001	<0.001	<0.001
	M	336.00 ^A^	324.00^B^	310.33^bC^	300.33^bC^				
	ML	331.00	328.00	325.67^a^	324.33^a^				
NDF (%)	CK	45.93^B^	47.90^aB^	50.93 ^aA^	52.70 ^aA^	0.165	<0.001	<0.001	0.606
	M	45.57^D^	47.57^aC^	49.87^aB^	52.23 ^aA^				
	ML	43.70 ^C^	43.90^bC^	46.77^bB^	48.97^bA^				
ADF (%)	CK	34.17^B^	35.10^B^	37.97 ^aA^	38.60 ^aA^	0.180	<0.001	<0.001	0.992
	M	33.73^B^	34.30^B^	36.77^acA^	38.00^acA^				
	ML	32.50^B^	32.77^B^	35.47^bcA^	36.27^bcA^				

Data are presented as means of three replicates. Values in the same row (A–D) or in the same column (a–c) with different superscripts are significantly different (p < 0.05).

^1^CK, control; M, molasses; ML, molasses and microbial inoculants.

^2^LAB, Lactic acid bacteria; AB, Aerobic bacteria; DM, Dry matter.

^3^A, Additive; D, ensilage time; A × D, the interaction between additive and ensilage time.

### Effect of ML on bacterial and fungal diversity in barley silage during aerobic stage

In this study, the operational taxonomic units and Chao 1 indices, which represented the richness of bacterial communities, had notably decreased in barley silage treated with ML at 2 and 5 d during aerobic stage (Table 3). Furthermore, the Shannon index of bacteria was not affected by M treatment only at 5 and 7 d during aerobic stage in barley silage, as compared with the CK silage. However, the Shannon index was lower in ML silage than that in the CK and M silages at 5 and 7 d during aerobic stage, suggesting that there was less bacterial diversity of barley silage in the ML silage than that in the CK and M silages.

**Table 3:** The bacterial and fungal alpha diversity of barely silage upon aerobic exposure.

	Aerobic exposure days	Treatments	Reads	OTUs	Shannon	Chao1	ACE	Coverage	PD^2^
Bacteria alpha diversity	Day 0	CK^1^	80985	90	1.918	98.600	101.629	1	9.079
		M	84447	76	2.190	95.610	93.211	1	6.667
		ML	80151	79	1.730	94.034	102.190	1	8.470
	Day 2	CK	83975	125	1.868	132.769	135.338	1	12.996
		M	80105	115	2.268	123.638	125.447	1	12.112
		ML	80148	74	1.446	90.503	95.080	1	7.036
	Day 5	CK	80234	106	2.927	114.310	117.325	1	12.392
		M	74289	143	2.928	151.942	157.347	1	14.543
		ML	80155	86	1.405	96.230	102.869	1	8.317
	Day 7	CK	84292	122	3.333	131.486	133.923	1	10.253
		M	77902	135	3.132	147.440	152.078	1	12.999
		ML	80095	119	1.518	133.612	138.871	1	13.361
Fungal alpha diversity	Day 0	CK	80235	379	4.077	438.041	440.218	0.998	23.048
		M	80210	425	4.356	463.494	470.875	0.998	31.927
		ML	80148	862	5.734	913.125	910.776	0.999	213.372
	Day 2	CK	80136	430	4.494	490.830	488.870	0.998	37.996
		M	80277	407	4.162	439.780	444.989	0.999	42.967
		ML	80150	666	5.092	764.486	797.796	0.998	189.794
	Day 5	CK	80238	319	3.997	354.980	346.732	0.999	16.503
		M	80327	412	4.135	428.787	430.988	0.999	18.565
		ML	80165	573	4.901	604.113	652.948	0.998	92.968
	Day 7	CK	80191	322	3.599	352.733	344.424	0.999	16.177
		M	80171	415	3.591	420.220	435.267	0.999	22.313
		ML	80201	461	4.283	498.437	508.309	0.999	45.203

^1^CK, control; M, molasses; ML, molasses and microbial inoculants.

^2^PD, PD_whole_tree.

Among the three groups, the operational taxonomic units, Chao 1, and Shannon indices of fungi were the highest in ML silage after 7d of exposure. Nevertheless, these indices decreased in the ML silage during aerobic stage. These findings show that the richness and diversity of fungal microbes had increased in barley silage treated with ML after aerobic exposure.

### Effect of ML on bacterial microbiomes in barley silage during aerobic stage

**Figure 2:**
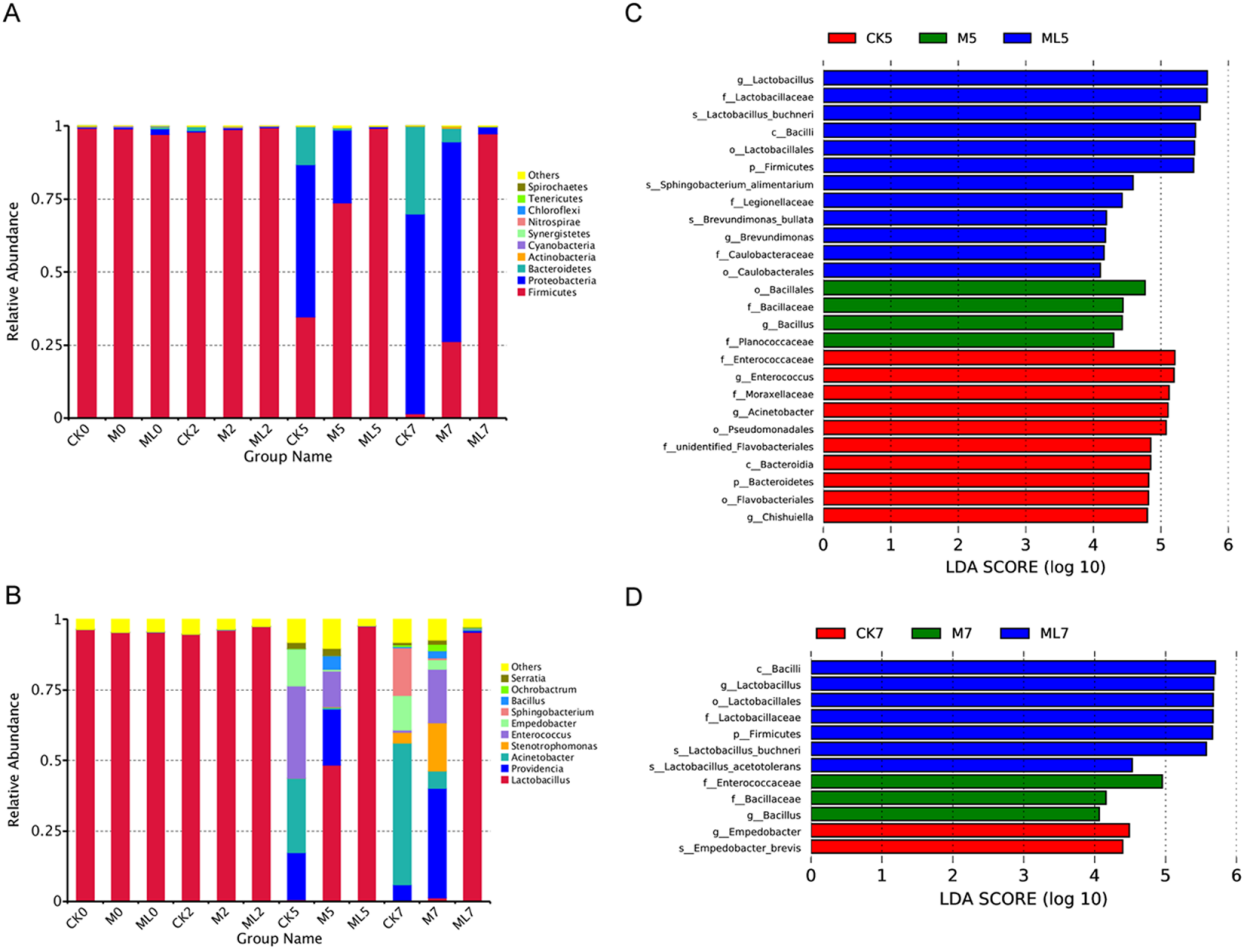
Bacterial community structure at the phylum (**A**) and genus (**B**) levels. Indicator bacteria in groups with LDA scores >4 at days 5 (**C**) and 7 (**D**) of aerobic exposure. Arabic numerals indicate the day of aerobic exposure. Abbreviations: CK, control; M, molasses; ML, molasses and microbial inoculants.

*Firmicutes* was the most abundant phyla with more than 97% relative abundance in ML silage during aerobic stage (Fig. 2A). However, the abundance of *Firmicutes* was decreased, whereas that of *Proteobacteria* was increased, with prolonged exposure in CK and M silages. Among the three groups, CK silage had the highest and the lowest abundances of *Proteobacteria* and *Firmicutes*, while the ML silage had the lowest and the highest abundances of *Proteobacteria* and *Firmicutes* at 5 and 7 d during the aerobic stage, respectively. In the CK, M, and ML groups, the abundances of *Firmicutes* at 7d of aerobic exposure were 1.544%, 26.291%, and 97.325%, respectively.

Changes at the genus level in bacterial community structures among the CK, M and ML silages during aerobic stage was summarized in Fig. 2B. Similar to *Firmicutes* at the phylum level, *Lactobacillus* was the dominant bacterial genus with a relative abundance of more than 95% in ML silage after aerobic exposure. Meanwhile, the abundance of *Lactobacillus* was high in the CK and M silages at 0 and 2d during aerobic stage. In CK silage at 5 d during aerobic stage, the dominant bacterial genera were *Enterococcus*, *Acinetobacter*, *Providencia*, and *Empedobacter* with relative abundances of 32.864%, 26.401%, 16.509%, and 13.024%, respectively, while that of *Lactobacillus* was only 0.708%. Furthermore, in M silage at 5 d during aerobic stage, *Lactobacillus*, *Providencia*, *Enterococcus*, *Acinetobacter, Empedobacter*, and *Bacillus* were the dominant bacterial genera with the abundances of 48.463%, 19.687%, 12.636%, 7.143%, 6.091%, and 4.755%, respectively. After 7 d of aerobic exposure, *Acinetobacter* was the prominent genus at 50.226% relative abundance in CK silage, which was accompanied by the emergence of *Sphingobacterium* with an abundance of up to 17.041% and a decrease in *Providencia* abundance to 5.717%. Meanwhile, in M silage, *Lactobacillus* abundance decreased from 48.463% at day 5 of exposure to 0.187% at day 7 of exposure. The abundances of *Providencia* and *Stenotrophomonas* in M silage increased to 38.866% and 17.031% at 7 d during aerobic stage, respectively, suggesting that they were the dominant genera.

Differences in bacterial community structures among the groups were identified by linear discriminant analysis (LDA) of effect size (LEfSe), which was used to calculate the relative abundances of bacterial genera in barley silage at 5 and 7 d during aerobic stage (Fig. 2C,D). LEfSe was used to identify the taxa that most likely explain the differences in bacterial community structures among the CK, M, and ML groups. The results revealed significant differences in LDA scores among the three groups. Enrichment of *L. buchneri* was significant in ML silage at 5 and 7 d during aerobic stage, suggesting that *L. buchneri* is a potential indicator of aerobic stability. Moreover, enrichment of *Bacillus* was significant in M silage at 5 and 7 d during aerobic stage. For *Empedobacter*, enrichment was significant in CK silage at 7 d during aerobic exposure, whereas for *Enterococcaceae* and *Acinetobacter*, enrichment was significant in CK silage at 5 d during aerobic exposure.

**Figure 3:**
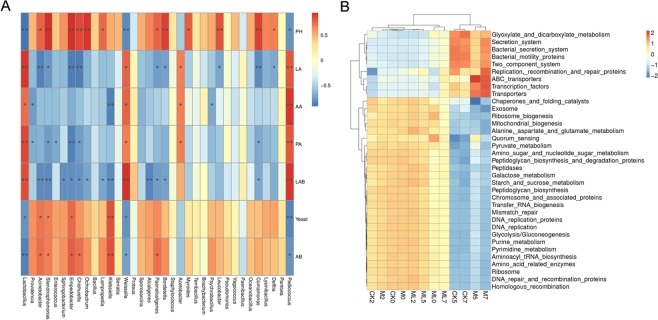
Spearman correlation heatmap of the top 35 genera of bacteria and fermentation properties (**A**). Heatmap of microbial function pathways (**B**). Arabic numerals indicate the day of aerobic exposure. Abbreviations: CK, control; M, molasses; ML, molasses and microbial inoculants.

The results of correlation analysis of bacterial communities, silage fermentation, and microbial counts of barley silage during aerobic stage are shown in Fig. 3A. When exposed to air for 7 days, *Lactobacillus*, *Weissella*, and *Pediococcus* were positively correlated with LA, AA, and PA concentrations, and LAB counts but negatively correlated with pH values and AB. *Acinetobacter* was positively correlated with pH values, as well as yeast and AB counts, but negatively correlated with LA concentrations and LAB counts. *Stenotrophomonas* and *Empedobacter* were positively correlated with pH values, as well as yeast and AB counts, but negatively correlated with LA and PA concentrations, and LAB counts.

Figure 3B shows a heatmap of microbial function pathways. A hierarchical cluster heatmap was generated to visualize the distribution of microbial communities in the CK, M, and ML groups. The heatmap results suggest that alanine aspartate and glutamate metabolism, pyruvate metabolism, amino sugar and nucleotide sugar metabolism, starch and sucrose metabolism, glycolysis, purine metabolism, pyrimidine metabolism, and aminoacyl tRNA biosynthesis, which are involved in amino acid metabolism, carbohydrate metabolism, nucleotide metabolism, and translation, respectively, were more abundant in the ML group than in the CK group. As compared with the ML group, the microbiota in the CK group had higher functional performance related to bacterial secretion systems, bacterial motility proteins, two-component systems, and adenosine triphosphate binding cassette transporters, which are involved in environmental information processing.

### Effect of ML on fungal microbiomes in barley silage after aerobic stage

**Figure 4:**
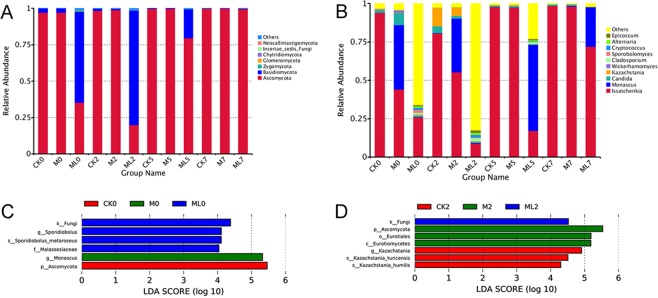
Fungal community structure at the phylum (**A**) and genus (**B**) levels. Indicator fungi in groups with LDA scores >4 at days 0 (**C**) and 2 (**D**) of aerobic exposure. Arabic numerals indicate the day of aerobic exposure. Abbreviations: CK, control; M, molasses; ML, molasses and microbial inoculants.

**Figure 5:**
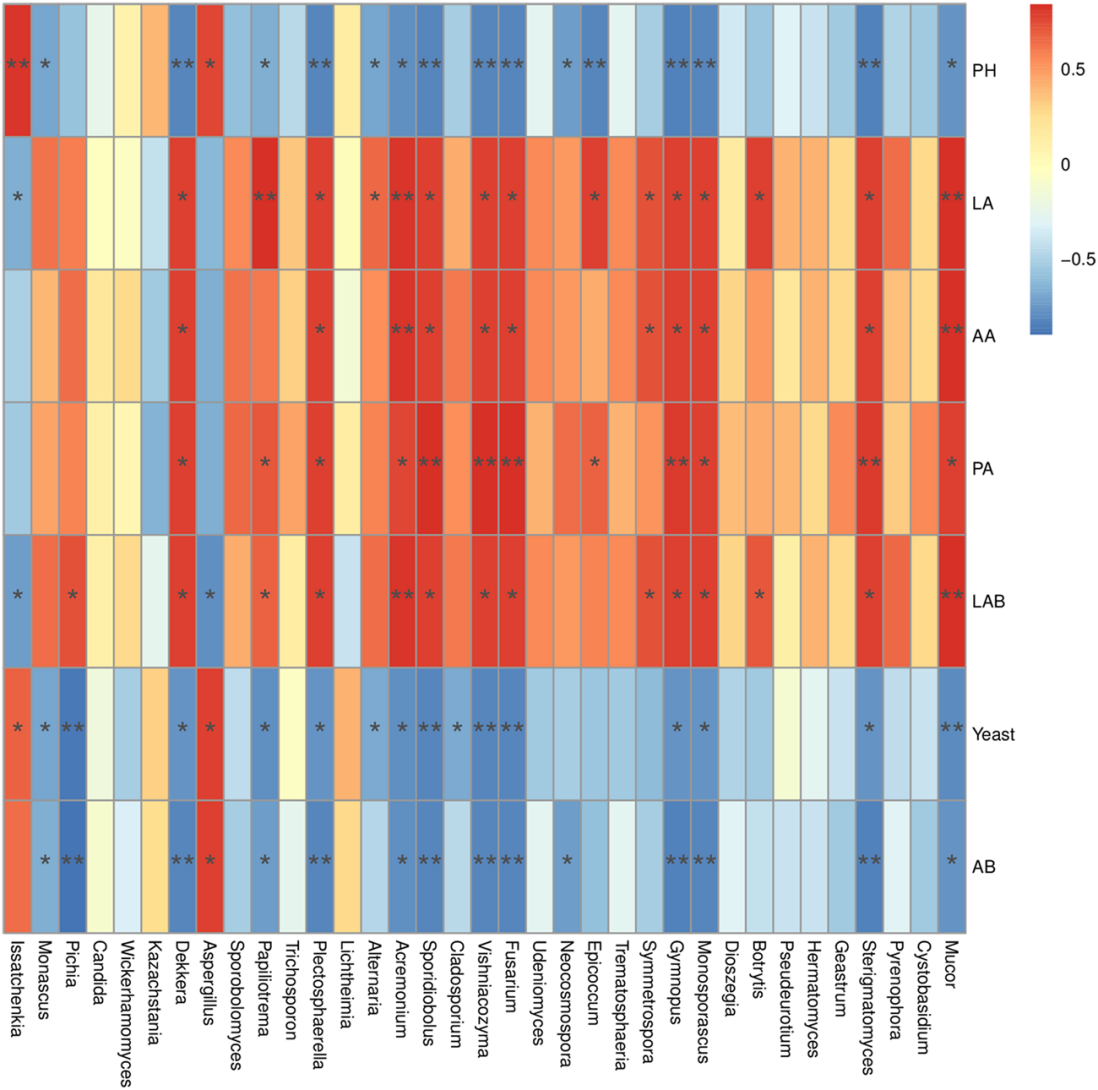
Spearman correlation heatmap of the top 35 genera of fungi and fermentation properties.

Fungal community composition at the phylum level in ML silage after aerobic stage was significantly different with CK silage (Fig. 4A). The members of *Ascomycota* phylum were more abundant in M and CK silages with an abundance of more than 97% during the aerobic stage. *Basidiomycota* in the ML silage was the preponderant fungi at the early stage of exposure, with abundances of 62.562% and 78.714% at 0 and 2 d, respectively, during the aerobic stage. *Ascomycota* was the most abundant group in the ML silage at 5 d of aerobic exposure. Figure 4B shows the differences in fungal community structures at the genus level among barley silages after the aerobic stage. The predominant fungal genera in the CK silage at 2 d during the aerobic stage were *Issatchenkia*, *Kazachstania*, and *Candida* with abundances of 80.580%, 12.055%, and 4.449% respectively. Meanwhile, *Issatchenkia*, *Monascus*, and *Kazachstania* were the major fungi in M treated silage at 2 d during aerobic stage with the abundances of 55.310%, 35.024%, and 5.487%, respectively. Compared with CK silage, the addition of M decreased the abundance of *Issatchenkia* and increased the abundance of *Monascus* and *Candida* at 0 and 2 d during aerobic stage. Furthermore, the abundances of *Issatchenkia*, *Candida*, and *Kazachstania* decreased in the ML silage after aerobic exposure, as compared with those of the CK and M silages. However, other genera (less than 1% sequenced) were the predominant fungi in ML silage at 2 d during aerobic stage at 82.5% abundance, followed by *Issatchenkia* (8.95%). This result was in accordance with fungal diversity of barley silage, which showed that ML silage had the greatest fungal diversity at 2 d during aerobic stage among the three groups, as shown in Table 3. Differences in fungal community structures among the groups was investigated using LEfSe analysis, which was used to calculate the relative abundance of fungi in barley silage at 0 and 2 d during aerobic stage. As shown in Fig. 4C,D, there were significant differences in fungal community structures between the ML and CK silages, which confirmed that significant enrichment of *Sporidiobolus* was a indicator of aerobic stability in the ML silage at 0 d during aerobic stage, although the abundance of *Kazachstania* in CK silage was significantly enriched at 2 d during aerobic stage. The relationship between the fungal community structure and fermentation quality (Fig. 5) was determined by canonical correlation analysis. *Issatchenkia* was positively correlated with pH values and yeast counts, but negatively correlated with LA concentrations and LAB counts. Meanwhile, *Sporidiobolus* was negatively correlated with pH values. The addition of M combined with microbial inoculants might have decreased the abundance of *Issatchenkia*, increased the abundance of others fungi (less than 1% sequence), decreased the pH value and content of yeast, and increased the contents LA and LAB of barley silage after aerobic exposure.

## Discussion

The pH value was one of the main factors that influenced the extent of fermentation and quality of silage. A low pH value of 3.94 or less at the terminal point of silage indicates good fermentation during ensiling and aerobic exposure. The decline in microbial growth, which may favor the growth of undesirable anaerobic organisms and subsequently decrease aerobic stability, was effectively inhibited in silage under low pH conditions^12,13^. Consistently, in this study, the pH values of barley silage prepared with ML were low, suggesting ML silage has high aerobic stability. During aerobic exposure, the pH values were always less than 4.0 in ML silage, while the pH value reached 4.85 in barley silage added with inoculants at day 7 of aerobic exposure^3^. The yeast and AB counts of the ML group were significantly lower than those of the CK and M groups during aerobic exposure. Meanwhile, the counts of yeast and AB in ML silage were also less than those of barley silage added with inoculants which reported by Liu *et al*.^3^ at day 5 and 7 of aerobic exposure. Yeasts, as primary initiators of aerobic spoilage in silage, assimilate lactate and degrade it into CO_2_ and water, which is accompanied with nutrient loss in silage exposed to air. As a result of lactate depletion by yeasts, pH increases, rendering silage more susceptible to spoilage^14^. Compare to LA, it is easier for yeasts to be inhibited by AA^15^. According to the report by Danner *et al*.^16^, silages with high concentrations of AA have low populations of yeasts and molds and high aerobic stability. Barley silage treated with ML had the highest AA content during the aerobic stage in this study. The lowest yeast population was observed at 7 d of aerobic exposure, indicating that the aerobic stability of ML silage was the highest among the groups. However, these results do not conform to those of Kim *et al*.^10^, who reported that silages treated with M and *L. plantarum* had the lowest AA content, the highest populations of yeasts, and the highest aerobic stabilities during the ensiling of silage.

After aerobic exposure, the diversity and richness of bacteria in ML silage were lower than that in CK and M silages, which might be attributed to the predominant roles of *L. plantarum*. This result is supported by the findings of a previous study conducted by Méndez-García *et al*.^17^, who indicated that limited microbial diversity was due to low pH values in acidic environments. A similar result was reported by Yang *et al*.^18^. Lower bacterial diversity and greater aerobic stability were also observed in silages with *L. buchneri* and PA than those of CK silages^19^. However, bacterial diversity did not decrease in M silage, as compared with CK silage, but was evidently reduced in ML silage during aerobic exposure in our study. Together, these results show that the richness and diversity of microbial communities in silage were affected by the addition of ML during aerobic exposure.

*Firmicutes* are gram-positive bacteria with genomes having low G + C contents and most degrade macromolecular compounds, such as cellulose, protein, and starch^20^. McGarvey *et al*. reported that *Firmicutes* and *Proteobacteria* were the dominant bacteria in alfalfa silage^21^. *Proteobacteria* is the largest phylum of bacteria and includes pathogenic bacteria, such as *Escherichia coli*, as well as *Vibrio* and *Helicobacter* spp., in addition to *Erwinia*, *Sphingomonas*, *Methylobacter*, *Pseudomonas*, and *Agrobacterium* spp.^22^. Romero *et al*. indicated a relatively high abundance of *Firmicutes* (99.8%) and a low abundance for *Proteobacteria* (0.07%) in wilted whole-crop oats ensiled for 217 days^20^. In this experiment, the bacterial community structure in silages changed substantially with prolonged exposure. Similar to the findings of Peng *et al*.^22^, in this study, the abundance of *Firmicutes* was significantly decreased, whereas that of *Proteobacteria* increased at 7 d during the aerobic stage in the CK and M silages. During aerobic exposure, the dominant phylum in ML silage was *Firmicutes*, with the abundance of >97%, which was significantly higher than that of barley silage added with inoculants. According to the findings of Liu *et al*.^3^, the dominant phylum in barley silage added with inoculants was *Firmicutes*, with the abundance of >99% at day 0 and 2 of aerobic exposure. However, *Firmicutes* and *Proteobacteria* were dominant in barley silage added with inoculants, with the abundance of 78.9% and 23.0% at day 5 of aerobic exposure, 20.8% and 69.2% at day 7 of aerobic exposure, respectively. According to a report by Xu *et al*.^23^, the abundance of *Firmicutes* predominated in spoiled silage after aerobic exposure, but was subsequently replaced by *Proteobacteria* and *Actinobacteria*.

*Lactobacillus* prevented aerobic spoilage due to the production of LA and the reduction of pH value^24^. In this study, the abundance of *Lactobacillus* predominated in ML silage throughout aerobic exposure, reaching more than 95%. At day 0 and 2 of aerobic exposure, *Lactobacillus* was dominant in CK silage and M silage, with the abundance of >94%. However, the abundance of *Lactobacillus* in M silage was only 48.2% and 1.2% at day 5 and 7 of aerobic exposure, respectively. Liu *et al*.^3^ reported that the abundance of *lactobacillus* for dominant genera in barley silage with inoculants kept more than 99% at day 0 and day 2 of aerobic exposure, but the abundance decreased to 71.4% and 11.6% at day 5 and 7 of aerobic exposure, respectively. Therefore, the addition of molasses combined with microbial inoculants could improve the structure of bacteria community which was predominated by *Lactobacillus* in barley silage during aerobic exposure. Pang *et al*.^25^ found that *Lactobacillus* was the prominent genus in sorghum, forage paddy rice, and alfalfa silages. M provides additional fermentable substrates for LAB, which promotes domination in the microbial communities of silage, thereby directing the metabolism to homofermentative LAB^26^. Li *et al*.^27^ reported that AA and PA might exert direct effects on bacterial physiology and indirect effects on the microbial community structure. In this study, the AA and PA concentrations in the ML group might be sufficiently high to effectively inhibit the growth of aerobic microorganisms that cause spoilage. With the addition of ML during the ensiling of barley silage, *Lactobacillus*, as beneficial bacteria, became the absolute dominant genus and competitively inhibited the growth of harmful bacteria, such as *Empedobacter* and *Acinetobacter*. The low bacterial diversity observed in ML silage, as indicated by the low Shannon index of 1.518, was a result of the high abundance of *Lactobacillus* species at 95.458% and low pH value (3.98) of barely silage after 7 d of exposure. *Lactobacillus buchneri* is a heterofermentative species that produces a variety of products and ferments LA into AA^2,28,29^. The conversion of LA to AA results in high concentrations of AA, which is regarded as an inhibitor of yeasts and molds. In this study, at 5 and 7 d during aerobic stage, LA and AA concentrations were higher in the ML silage than the CK and M silages. Hence, barley silage ensiling with ML had the best aerobic stability among the three groups. This result is possibly due to *L. buchneri* from ML silage, which plays an important role in pH, fermentation products, and microbial community dynamics during aerobic exposure.

Graf *et al*.^30^ deduced that the proliferation of bacilli normally occurs in the later period of aerobic spoilage, as indicated by the detection of *Bacillus* spores at the outermost layers of grass and maize silages. According to Liu *et al*.^31^, *Stenotrophomonas* possibly caused aerobic deterioration of corn straw silage. *Acinetobacter* are aerobic non-fermenting bacteria that exist in various environments except silage^32^. According to Liu *et al*.^31^, AB in corn straw silage upon aerobic exposure mainly comprises *Acinetobacter, Stenotrophomonas*, and *Bacillus*, which possibly cause aerobic deterioration of corn straw silage. In the present study, these aerobic bacteria were positively correlated with pH values, as well as yeast and AB counts, and promoted the growth of spoilage microorganisms.

As compared with the ML group, the microbiota in the CK group had higher functional performance related to bacterial secretion systems, bacterial motility proteins, two-component systems, and adenosine triphosphate binding cassette transporters, which are involved in environmental information processing. This result is in agreement with those of Keshri *et al*.^33^, who reported that silage treated with *L. buchneri*, which was stable after aerobic exposure, differed from untreated silage at day 90 in terms of three pathways; namely, base-excision repair, pyruvate metabolism, and transcription machinery. Notably, a high degree of pyruvate metabolism was also observed in LAB-supplement barley silage, suggesting a possible reason for the enhancement of silage stability^3^. Amino acids were metabolized by the dominant microflora in *L. plantarum* supplement and untreated silages during the ensiling^33^. The addition of *L. buchneri* increased the accumulation of polyols and free amino acids, which was possibly due to the unique metabolic pathways of sugar fermentation and amino acid biosynthesis executed by *L. buchneri*^34^. In the present study, the changes in the carbohydrate and amino acid metabolic pathways may have contributed to the inhibition of aerobic spoilage in ML silage. As compared with the CK silage, the higher abundances of carbohydrates and amino acids were responsible for the improved aerobic stability of LAB-inoculated barley silages^3^.

The members of *Ascomycota* phylum were more abundant in M and CK silages with an abundance of more than 97% during the aerobic stage. These results were similar to the findings of Romero *et al*.^20^, who reported that *Ascomycota* was the most abundant group, and *Basidiomycota* was the second most abundant in oat silage. In our study, *Basidiomycota* in the ML silage was the preponderant fungi at the early stage of exposure during the aerobic stage. *Ascomycota* was the most abundant group in the ML silage at 5 d of aerobic exposure, which was accompanied with a decrease in the abundance of *Basidiomycota*, suggesting that the fungal community structure was significantly modified by ML treatment at the early stage of aerobic exposure. Yeasts, such as *Issatchenkia*, *Candida*, and *Saccharomyce*s spp, are considered basic initiators of aerobic spoilage in barley silage^35^. The abundances of *Issatchenkia*, *Candida*, and *Kazachstania* decreased in the ML silage after aerobic exposure, as compared with those of the CK and M silages, suggesting the addition of ML affected fungal community composition. *Issatchenkia* was the main microorganism causing aerobic deterioration of corn and barley silages^35,36^. Liu *et al*.^3^, indicated the addition of inoculants could decrease the abundances of *Issatchenkia* at day 0 of aerobic exposure, but not affect the abundances of *Issatchenkia* at day 2, 5 and 7 of aerobic exposure. Our results suggested the addition of molasses combined with microbial inoculants could affect fungal community composition and decrease the abundances of *Issatchenkia* in barley silage, however, single addition of inoculants had little effect on fungal community composition during aerobic exposure. The abundance of harmful microorganisms, such as aerobic bacteria including *Acinetobacter*, *Providencia*, *Bacillus*, and yeasts including *Issatchenkia*, *Candida*, and *Kazachstania*, were inhibited in the ML silage. *Sporidiobolus* can be exploited for biotechnological production of β-carotene using conventional fed-batch fermentation^37^. *Sporidiobolus* and *Sporobolomyces* produce carotenoids, which are effective antioxidants having potential antimicrobial properties^38^. In addition, similar results reported by Dolci *et al*. showed that the dominant yeast species in corn silage was *Kazachstania exigua* after exposure to air, as determined by denaturing gradient gel electrophoresis profiles of fungal DNA and RNA^39^. According to Lu *et al*., *Kazachstania unispora* was cardinal among the dominant species of yeasts in aerobically deteriorating corn silage^40^.

## Conclusion

ML silages remained aerobically stable for more than 216 h with stable pH values and LA contents. Bacterial diversity was reduced, while fungal diversity was increased in the ML silage during aerobic exposure. The abundance of *Lactobacillus* was enriched, while that of harmful microorganisms, such as aerobic bacterial including *Acinetobacter*, *Providencia*, *Bacillus*, and yeasts including *Issatchenkia*, *Candida*, and *Kazachstania*, were lowest in the ML silage. The addition of ML could increase AA content, reduce yeast and AB counts, change bacterial and fungal microbes, improve fermentation quality, and reduce aerobic spoilage of barley silage.

## Methods

### Preparation of barley silage

Barley was harvested from a farm located in Yancheng, Jiangsu, China (33°19′ N, 120°45′ E) in May 2017. Barley was chopped using fully automatic harvester. We used inoculants and molasses as additives for barley silage. The inoculants were prepared by our laboratory and comprised *L. plantarum*, *L. casei*, and *L. buchneri* (2:1:1)^3^, which were isolated form ryegrass silage, rice straw silage, corn silage, respectively. The NCBI accession number of inoculants were MK106013, MK106014, MK106015, respectively. The combined inoculants were added at 5 × 10^5^ colony forming units (CFU) g^−1^ of fresh material (FM). The chopped barley was mixed and divided into equal portions for three treatments comprising no additive (CK group), 2 g of M per 100 g of FM (M group), or 2 g of M per 100 g of FM + inoculants (ML group). After thorough mixing, barley was ensiled in 1 L silos.

After 60 days of ensiling, silos were opened. Fermentation quality, microbial counts, chemical compositions, bacterial and fungal community structures were analyzed at 0, 2, 5, and 7 d during aerobic exposure. The aerobic stability was determined using a previously described method^41^.

### Fermentation quality and chemical composition

The pH value and organic acid concentrations were determined by homogenizing 20 g of silage in 180 mL of sterilized water at 4 °C for 24 h and then filtrating the homogenate through four layers of cheesecloth. The pH of the filtrate was measured using a glass electrode pH meter (Mettler-Toledo AG, Zurich, Switzerland). LA, acetic acid (AA), and propionic acid (PA) and ethanol were determined by high performance liquid chromatography (Agilent 1260; Agilent Technologies, Santa Clara, CA, USA). The analytical conditions were as follows: column, Carbomix® H-NP5 (Sepax Technologies, Inc., Newark, DE, USA); oven temperature, 50 °C; mobile phase, 2.5-mM H_2_SO_4_; flow rate, 0.5 mL/min). Approximately 100 g of each sample was dried at 55 °C for 48 h and weighed to determine the dry matter content of barley silage. Both neutral and acid detergent fibers were analyzed using the method described by Van Soest *et al*.^42^.

### Microbial counts

Twenty grams of each sample in 180 mL of physiological saline was blended and serially diluted. Microbial counts of LAB, yeasts, and aerobic bacteria (AB) were measured according to the study of Liu *et al*.^3^. The culture medium used were lactobacilli de Man, Rogosa, Sharpe agar (CM361; Oxoid Ltd.), potato dextrose agar (CM0139; Oxoid Ltd.), and nutrient agar (CM1160; Oxoid Ltd.). Lactic acid bacteria were incubated at 30 °C for 48 h under anaerobic conditions, whereas yeasts and aerobic bacteria were incubated at 30 °C for 24 h. Colonies were counted in CFU/g FM^−1^ as viable numbers of microorganisms.

### Bacterial and fungal community

Total DNA was extracted using the PowerSoil® DNA Isolation Kit (MO BIO Laboratories, Carlsbad, CA, USA) in accordance with the manufacturer’s protocol. The V3-V4 region of bacterial 16S rDNA and the fungal internal transcribed spacer 1 region were amplified, sequenced, and analyzed according to the methods described by Liu *et al*. and Zhang *et al*.^3,43^. The raw reads were deposited in the European Nucleotide Archive (ENA) database under the accession number ERP118410 and ERP118523.

### Statistical analysis

Fermentation quality, microbial counts, nutritional value were analyzed by two-way analysis of variance for a 3 × 4 (treatment × storage period)-factorial arrangement of treatments and compared with Tukey’s test using IBM SPSS Statistics for Windows, version 19.0. (IBM Corporation, Armonk, NY, USA). A probability (*p*) value of <0.05 was considered statistically significant.

## References

[1] Muck RE (2018). Silage review: Recent advances and future uses of silage additives. J. Dairy Sci..

[2] Silva LD (2018). Effects of *Lactobacillus buchneri* isolated from tropical maize silage on fermentation and aerobic stability of maize and sugarcane silages. Grass. Forage. Sci..

[3] Liu BY (2019). Dynamics of a microbial community during ensiling and upon aerobic exposure in lactic acid bacteria inoculation-treated and untreated barley silages. Bioresour Technol..

[4] Guo G, Yuan XJ, Li LX, Wen AY, Shao T (2014). Effects of fibrolytic enzymes, molasses and lactic acid bacteria on fermentation quality of mixed silage of corn and hulless-barely straw in the Tibetan Plateau. Grassl. Sci..

[5] Bilal MQ (2009). Effect of molasses and corn as silage additives on the characteristics of Mott Dwarf elephant grass silage at different fermentation periods. Pak. Vet. J..

[6] Qiu XY, Guo G, Yuan XJ, Shao T (2013). Effects of adding acetic acid and molasses on fermentation quality and aerobic stability of total mixed ration silage prepared with hulless barley straw in Tibet. Grassl. Sci..

[7] Yuan XJ (2015). Effects of ethanol, molasses and *Lactobacillus plantarum* on fermentation characteristics and aerobic stability of total mixed ration silages. Grass. Forage. Sci..

[8] Ni KK (2017). Effects of lactic acid bacteria and molasses additives on the microbial community and fermentation quality of soybean silage. Bioresour Technol..

[9] Hashemzadeh-Cigari F (2014). Interactive effects of molasses by homofermentative and heterofermentative inoculants on fermentation quality, nitrogen fractionation, nutritive value and aerobic stability of wilted alfalfa *(Medicago sativa L)* silage. J. Anim. Physiol. An. N..

[10] Kim JS (2016). Effect of microbial inoculant or molasses on fermentative quality and aerobic stability of sawdust-based spent mushroom substrate. Bioresour Technol..

[11] Wang C (2019). Fermentation quality and microbial community of alfalfa and stylo silage mixed with *Moringa oleifera* leaves. Bioresour Technol..

[12] He LW (2020). Improving the quality of rice straw silage with *Moringa oleifera* leaves and propionic acid: fermentation, nutrition, aerobic stability and microbial communities. Bioresour Technol..

[13] Tabacco E, Righi F, Quarantelli A, Borreani G (2011). Dry matter and nutritional losses during aerobic deterioration of corn and sorghum silages as influenced by different lactic acid bacteria inocula. J. Dairy Sci..

[14] Kleinschmit DH, Schmidt RJ, Kung L (2005). The effects of various antifungal additives on the fermentation and aerobic stability of corn silage. J. Dairy Sci..

[15] Wilkinson JM, Davies DR (2013). The aerobic stability of silage: key findings and recent developments. Grass. Forage. Sci..

[16] Danner H, Holzer M, Mayrhuber E, Braun R (2003). Acetic acid increases stability of silage under aerobic conditions. Appl. Environ. Microbiol..

[17] Méndez-García C (2015). Microbial diversity and metabolic networks in acid mine drainage habitats. Front. Microbiol..

[18] Yang LL, Yuan XJ, Li JF, Dong ZH, Shao T (2019). Dynamics of microbial community and fermentation quality during ensiling of sterile and nonsterile alfalfa with or without *Lactobacillus plantarum* inoculant. Bioresour Technol..

[19] Ogunade IM (2017). Fate of Escherichia coli O157:H7 and bacterial diversity in corn silage contaminated with the pathogen and treated with chemical or microbial additives. J. Dairy Sci..

[20] Romero JJ (2017). Laboratory silo type and inoculation effects on nutritional composition, fermentation, and bacterial and fungal communities of oat silage. J. Dairy Sci..

[21] McGarvey JA (2013). Bacterial population dynamics during the ensiling of *Medicago sativa* (alfalfa) and subsequent exposure to air. J. Appl. Microbiol..

[22] Peng K (2018). Condensed tannins affect bacterial and fungal microbiomes and mycotoxin production during ensiling and upon aerobic exposure. Appl. Environ. Microb..

[23] Xu SW (2019). Impact of *Saccharomyces cerevisiae* and *Lactobacillus buchneri* on microbial communities during ensiling and aerobic spoilage of corn silage. J. Anim. Sci..

[24] Wang, Y. *et al*. Dynamics of Bacterial Community and Fermentation Quality during Ensiling of Wilted and Unwilted *Moringa oleifera* Leaf Silage with or without Lactic Acid Bacterial Inoculants. *mSphere*. **4**, e00341–19, 10.1128/mSphere.00341-19 (2019b). 10.1128/mSphere.00341-19 PMC6686226 31391277

[25] Pang H (2011). Natural populations of lactic acid bacteria associated with silage fermentation as determined by phenotype, 16S ribosomal RNA and recA gene analysis. Syst. Appl. Microbiol..

[26] Cao Y, Takahashi T, Horiguchi KI, Yoshida N (2010). Effect of adding lactic acid bacteria and molasses on fermentation quality and in vitro ruminal digestion of total mixed ration silage prepared with whole crop rice. Grassl. Sci..

[27] Li P (2019). Silage fermentation and bacterial community of bur clover, annual ryegrass and their mixtures prepared with microbial inoculant and chemical additive. Anim. Feed Sci. Tech..

[28] Mari LJ, Schmidt RJ, Nussio LG, Hallada CM, Kung L (2009). Short communication: an evaluation of the effectiveness of *Lactobacillus buchneri* 40788 to alter fermentation and improve the aerobic stability of corn silage in farm silos. J. Dairy Sci..

[29] Schmidt RJ, Kung L (2010). The effects of *Lactobacillus buchneri* with or without a homolactic bacterium on the fermentation and aerobic stability of corn silages made at different locations. J. Dairy Sci..

[30] Graf K, Ulrich A, Idler C, Klocke M (2016). Bacterial community dynamics during ensiling of perennial ryegrass at two compaction levels monitored by terminal restriction fragment length polymorphism. J. Appl. Microbiol..

[31] Liu QH, Shao T, Zhang JG (2013). Determination of aerobic deterioration of corn stalk silage caused by aerobic bacteria. Anim. Feed Sci. Tech..

[32] Li Y, Nishino N (2011). Effects of inoculation of *Lactobacillus rhamnosus* and *Lactobacillus buchneri* on fermentation, aerobic stability and microbial communities in whole crop corn silage. Grassl. Sci..

[33] Keshri J (2019). Bacterial dynamics of wheat silage. Front. Microbiol..

[34] Guo XS (2018). Profiling of metabolome and bacterial community dynamics in ensiled Medicago sativa inoculated without or with *Lactobacillus plantarum* or *Lactobacillus buchneri*. Sci. Rep..

[35] Inglis GD, Yanke LJ, Kawchuk LM, McAllister TA (1999). The influence of bacterial inoculants on the microbial ecology of aerobic spoilage of barley silage. Can. J. Anim. Sci..

[36] Santos MC (2017). Identification of the major yeasts isolated from high moisture corn and corn silages in the United States using genetic and biochemical methods. J. Dairy Sci..

[37] Chaiyaso T, Manowattana A (2018). Enhancement of carotenoids and lipids production by oleaginous red yeast *Sporidiobolus pararoseus* KM281507. Prep. Biochem. Biotech..

[38] Fiedor J, Burda K (2014). Potential role of carotenoids as antioxidants in human health and disease. Nutrients..

[39] Dolci P, Tabacco E, Cocolin L, Borreani G (2011). Microbial dynamics during aerobic exposure of corn silage stored under oxygen barrier or polyethylene films. Appl. Environ. Microbiol..

[40] Lu HZ, Cai Y, Wu ZW, Jia JH, Bai FY (2004). *Kazachstania aerobia* sp. nov., an ascomycetous yeast species from aerobically deteriorating corn silage. Int. J. Syst. Evol. Microbiol..

[41] Yuan XJ (2018). Effects of four short-chain fatty acids or salts on fermentation characteristics and aerobic stability of alfalfa (*Medicago sativa* L.) silage. J. Sci. Food. Agric..

[42] Van Soest PJ, Robertson JB, Lewis BA (1991). Methods for dietary fibre, neutral detergent fibre, and non-starch polysaccharides in relation to animal nutrition. J. Dairy Sci..

[43] Zhang, L. *et al*. Analysis of the correlation between bacteria and fungi in sugarcane tops silage prior to and after aerobic exposure. *Bioresour Technol*. **291**, 121835, 10.1016/j.biortech.2019.121835 (2019). 10.1016/j.biortech.2019.121835 31352166

